# Miniaturized Biosensors Based on Lanthanide-Doped Upconversion Polymeric Nanofibers

**DOI:** 10.3390/bios14030116

**Published:** 2024-02-21

**Authors:** Neha Dubey, Sudeshna Chandra

**Affiliations:** 1 Department of Chemistry, Sunandan Divatia School of Science, SVKM’s NMIMS (Deemed to be) University, V.L. Mehta Road, Vile Parle (West), Mumbai 400056, India; neha.dubeyvisfac@svkmmumbai.onmicrosoft.com; 2 Hanse-Wissenschaftskolleg—Institute for Advanced Study (HWK), Lehmkuhlenbusch 4, 27753 Delmenhorst, Germany

**Keywords:** upconversion nanoparticles, polymeric nanofibers, biosensors, upconversion luminescence, lanthanides

## Abstract

Electrospun nanofibers possess a large surface area and a three-dimensional porous network that makes them a perfect material for embedding functional nanoparticles for diverse applications. Herein, we report the trends in embedding upconversion nanoparticles (UCNPs) in polymeric nanofibers for making an advanced miniaturized (bio)analytical device. UCNPs have the benefits of several optical properties, like near-infrared excitation, anti-Stokes emission over a wide range from UV to NIR, narrow emission bands, an extended lifespan, and photostability. The luminescence of UCNPs can be regulated using different lanthanide elements and can be used for sensing and tracking physical processes in biological systems. We foresee that a UCNP-based nanofiber sensing platform will open opportunities in developing cost-effective, miniaturized, portable and user-friendly point-of-care sensing device for monitoring (bio)analytical processes. Major challenges in developing microfluidic (bio)analytical systems based on UCNPs@nanofibers have been reviewed and presented.

## 1. Introduction

Lab-on-a-chip, frequently referred to as micro-fabricated, (bio)analytical devices provide an efficient interface for multiple physiologically significant chemical evaluations. The creation and application of microfluidic-based (bio)analytical techniques encompasses a variety from both established ideas and innovations such as micromachining, microlithography, the field of nanotechnology, and micro-electromechanical systems [1]. Mechanism-based (bio)analytical methods (MBBTs) can reveal the presence of individual molecules and compounds that are toxic and harmful. The gene induction assays, enzyme inhibition assays, receptor assays, and immunoassays are included in MBBTs. The initial interaction of a chemical species with a biological target and their subsequent transformation into a specific signal forms the basis of (bio)analytical techniques. For instance, the chemical binding of a ligand to a biological receptor may be the first step in the chemical–biological interaction. It would then be feasible to quantify the actual ligand–receptor association by determining the gene that encodes luciferase expression in a cell sensor gene bioassay. The (bio)analytical techniques are also preferred over conventional equipment like high-performance liquid chromatography (HPLC) and liquid chromatography tandem mass spectrometry (LC-MS/MS) because of the requirement for smaller sample volumes, quicker response time, and economical benefits.

Microfluidics is an emerging technology that has been regarded as crucial in various fields, from material science to biomedical engineering. Bioluminescence-based optical biosensors are used to analyze variations in the bioluminescence pattern of a luminous bacteria-incubated sample in comparison to a control sample [2,3]. By absorbing low-energy photons in the near-infrared (NIR) range, lanthanide-doped upconversion nanoparticles (UCNPs) can exhibit high-energy photon emission in the visible spectrum. The UCNPs’ high-energy luminescent emission has lengthy decay times, a minimum background signal, and remarkably crisp optical characteristics. The energy associated with these excited states further splits under the crystal field once it is entwined in massive crystals and nanostructures, establishing an array of modes with several distant energies. On the anatomical scale, the physical mechanisms causing upconversion in nanoparticles are the same as those in bulk crystals; however, there are special concerns in the realm of nanoparticles regarding their overall efficiency and other ensemble effects. The 4f-4f or 4f-5d transitions underlie the lanthanides’ (Ln^3+^) distinctive emission [4], which on excitation produces strong emissions in the NIR, visible, and UV regions, thus acting as optically active centers. Lanthanide-doped UCNPs can be regarded as host–guest systems where Ln^3+^ are guest molecules that occupy the host lattice. Host materials like NaYF_4_, NaGdF_4_, CaF_2,_ etc., [5,6,7,8] are generally used to hold the lanthanides (sensitizer and activator ions) within a proper distance to generate intense NIR-to-visible upconversion (UC) fluorescence. When doped with sensitizers (Yb^3+^) and activators (Er^3+^, Tm^3+^, Ho^3+^), the host can emit strong green and red light. Although the photon upconversion mechanism in lanthanide-doped nanoparticles is generally the same as in bulk material, it has been demonstrated that certain factors linked to the surface and size of the UCNPs have significant implications [9]. Since the 4f electrons are suitably localized, quantum confinement is not predicted to affect the energy levels in lanthanide ions; nonetheless, other phenomena have been demonstrated to have significant influence on the emission spectra and efficiency of UCNPs. The conflict between radiative and non-radiative relaxation makes the phonon density of states a crucial consideration. Furthermore, phonon-assisted processes play a crucial role in bringing the f orbitals’ energy states into range for energy transfer to take place. Although low-frequency vibrations are not found in the spectrum in nanocrystals/nanomaterials, the phonon band diminishes to a distinct collection of states. The repercussions of dimensionality are convoluted because of the competition between non-radiative relaxation, which shortens the durations of excited states, and phonon-assistance, which enhances the energy transfer. The color and efficiency of luminescence are also influenced by surface-related factors. Large vibrational energy levels of surface ligands on nanocrystals can greatly enhance phonon-assisted actions [10].

Therefore, research has been attracted to their applications in bioimaging-guided disease monitoring, photodynamic therapy (PDT), (bio)analytical sensing, photovoltaics, and so on [11,12,13]. Obtaining quick, consistent, and reliable analytical signals is a crucial component of a sensing platform that qualifies for bioassay in bio(analytical) sensing. Most fluorescent dyes have modest luminous life spans, light bleaching, auto-fluorescence in living tissues, as well as moderate Stokes shifts, which limit their potential in biosensing and biochemical tests. However, UCNPs can circumvent these limitations due to their unique capacity to absorb NIR light and display compact emission bands at an inferior wavelength. This can lessen light dispersion, enable deep tissue penetration without inducing autofluorescence, and can have an extended luminescence lifespan. When developing assays, UCNPs’ ruggedness and broad chemical range provide a variety of choices, including the recognition of ions in the inside region of the cells and analytes as well as biomarkers [14]. Upconversion nanoparticles based on a crystalline host lattice (in most cases hexagonal NaYF_4_) and doped with different combinations of lanthanide ions diverge from other nanoscale luminescent probes mainly in using infrared excitation waves, as they frequently cause biological tissues and polymeric materials to become transparent in the infrared spectrum, low auto-fluorescence, and high signal-to-noise ratios. The numerous long-lived electronic states of lanthanide ions are sequentially absorbed by several photons, converting the NIR light into visible and UV light into a nonlinear process. This method permits lower excitation intensity because it increases the likelihood of attaining higher excited states than two-photon excitation of dyes [15].

**Figure 1:**
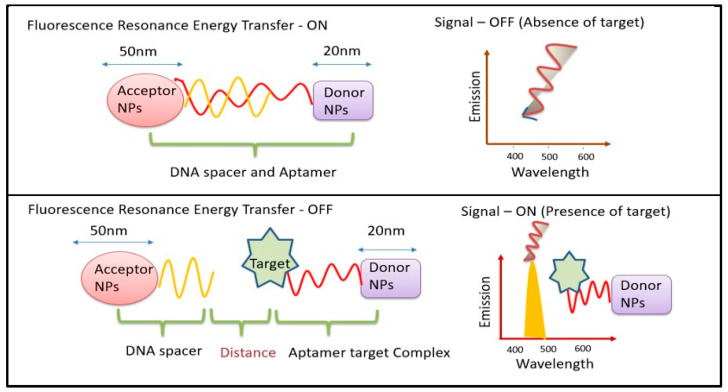
Schematic diagram for mechanism of FRET biosensors.

The two main mechanisms used by UCNP-using biosensors are fluorescence and FRET. One of the three methods—fluorescence enhancement (turning on), fluorescence quenching (turning off), or fluorescence resonance energy transfer (FRET)—is employed by fluorescence-based biosensors. Generally, turn/switch/signal-ON or turn/switch/signal-OFF FRET tests are categorized. FRET can occur when an acceptor and a donor molecule are brought close to one another under specific circumstances, as shown in (Figure 1). Numerous techniques can be used to achieve FRET detection and quantification. The phenomenon can be observed by exciting a specimen that contains molecules of both the donor and the acceptor, with light emitted at wavelengths centered near the acceptor’s emission maximum. The two signals can be used for a ratiometric analysis since FRET can result in both an increase in the acceptor’s fluorescence and a decrease in the donor molecule’s fluorescence. The benefit of this method is that it takes interaction measurement independent of the absolute concentration of the sensor. Since not all acceptor moieties are fluorescent, they can be employed to dampen fluorescence. In these situations, there would be a decrease in the signal from interactions that cause a fluorescent donor molecule to become near to such a molecule. As UCNPs have an electron relaxation period in the temporal frame, UCNP-based FRET systems are commonly referred to in the literature as luminescent resonance energy transfer (LRET). The fact that the stimulus needed to activate the donor must be outside of the environment’s absorption range makes UCNPs excellent options for biological-sample detection [16]. An increasing number of UCNPs have been integrated onto nanofibers in recent years to serve as both on- and off-chip transducers in biosensors.

A one-dimensional nanostructure with a diameter usually between 10 and 100 nm makes up a nanofiber. When loaded with biorecognition molecules, nanofibers with a high surface-to-volume ratio can offer a large sensing surface without removing a significant amount of sample volume [17]. An emerging field of study for biomedical applications is the controlled manufacturing of well-defined fibrillar nano-structured composites, which may be tailored to acquire desired mechanical and chemical properties that can also resemble a cellular matrix. Until now, the majority of UCNP-based sensor devices and applications have been intensity-based; variations in particle concentration result in unfavorable uncertainties and a larger detection limit. PMMA, polystyrene nanofibers, PVP poly(ε-caprolactone), and TiO_2_ nanofibers have all effectively incorporated UCNPs [18,19]. An oxygen sensor based on the ruthenium complex’s spectrum overlap with the emission of Tm^3+^ doped UCNPs embedded in polysulfone nanofibers was described by Presley et al. [20], and Fu et al. investigated a microRNA detection technique. Light scattering effects on the surface are enhanced when nanomaterials, such as luminous nanofibers, are incorporated into microfluidic channels, which hinders an adequate signal response [21].

We have covered in depth the effects of UCNP embedding on luminescence efficiency in this review, along with the developments and difficulties associated with UCNP@nanofibers, particularly in (bio)analytical devices. We have also focused on the fundamental process that UCNPs use for these (bio)analytical devices. We have examined both conventional and novel approaches to circumvent the quenching effect of UCNP@nanofibers.

### 1.1. Impact of Morphology of UCNP@nanofibers on Luminescence Efficiency

**Figure 2:**
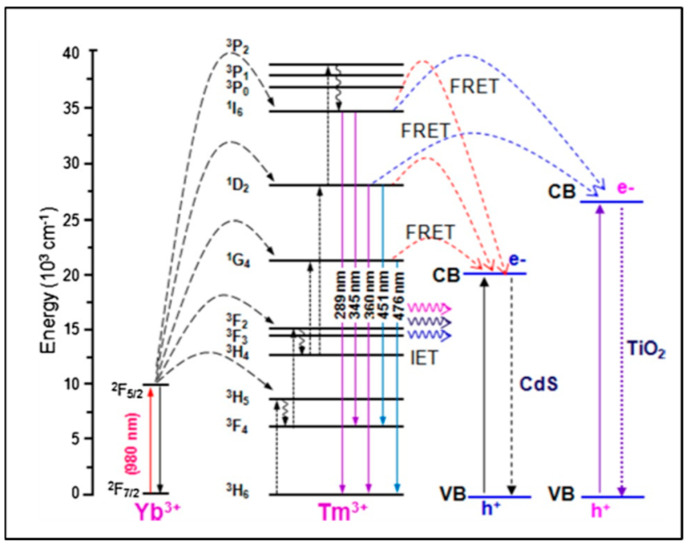
Irradiative energy transfer (IET) and non - irradiative energy transfer (FRET) processes (Adapted from Ref. [26]).

The design and morphology of UCNP@nanofibers have a significant effect on plasmon-enhanced luminescence efficiency which depends on a complex interplay between different dopant ions and host lattices. Electrospinning has been considered as one of the best techniques for fabricating both organic and inorganic nanofibers mainly because the nanoparticles can be easily combined with the fibers to form composite nanofibers. Mainly, the nanoparticles are either doped inside the nanofibers or loaded on their surface. These nanofibers may range within tens to hundreds of nanometers in diameter. Various other parameters that contribute to the formation of nanofibers are the electrospinning solution (polymer blend/nanoparticles/biomolecules), electrical conductivity and rheological properties of the blend, and processing conditions like applied voltage, flow rate of the blend. Yang and colleagues in 2012 reported, for the first time, the upconverted fluorescence of lanthanide-doped YOF nanofibers [22]. Varying the composition of the lanthanides and at 980 nm excitation, blue and red emissions were obtained using YOF: Yb^3+^,Tm^3+^, and YOF:Yb^3+^,Er^3+^, respectively. The morphology and size of the Ln^3+^-doped YOF nanofibers were governed using electrospinning parameters like voltage, distance between the tip and collector, relative humidity, and, most importantly, the mass ratio of PVP in the solution. An increase in the diameter of the nanofibers was observed with the increase in the mass ratio of precursor/PVP in solution. Large-scale free-standing and flexible uniform NaYF_4_:Yb/Tm@NaYF_4_@TiO_2_ nanofibers were reported by Qian and colleagues [23] in which water dispersible UCNPs were first assembled into a polyvinyl pyrrolidone (PVP)/tetrabutyl titanate (TBT) matrix with electrospinning and then calcined at a high temperature to form the TiO_2_-based nanofibers. The TiO_2_ nanofibers were activated with the UCNPs via a non-irradiative resonant energy transfer process (FRET) upon excitation with the near-IR light. The energy conversion efficiency between the donor and the acceptor chromophore depends on the structure and distance between the chromophores [24]. The energy transfer occurs through charge–charge interaction between the diploes of the oscillating donor and acceptor molecules that are in close proximity (~1 to 10 nm). In addition to the distance, a favorable orientation of the transition dipole moments of donor and acceptor molecules are also required for an effective FRET. An overlap of lanthanide PL (photo-luminescence) emission and the FRET acceptor absorption spectra can be applied for multiplexed bio-analytical analyses. To enable full-spectrum absorption of solar energy (280 to 800 nm), the UCNPs@TiO_2_ nanofibers were embedded with semiconductor CdS nanoparticles which caused efficient transfer of the NIR photon energy to the CdS nanoparticles and TiO_2_ using irradiative energy-transfer (IET) and FRET processes (Figure 2) [25]. With respect to the shape of the host matrix, hexagonal NaYF_4_ has been reported to be a better upconverting host lattice than the cubic lattice NaYF_4_. Further, core–shell UCNPs are considered to be the best NIR-to-UV UCNPs, particularly with a concentration range of NaYF_4_:Yb(20–30%)/Tm(0.2–0.5%)@NaYF_4_ [26]. The low doping ratio of Tm prevented cross-relaxations. However, the choice of emitting UCNPs is limited to NaYF_4_:Yb/Tm@NaYF_4_ with low NIR-to-UV efficiency for application in live systems where quenching of the emission is induced by molecules containing hydroxyl (-OH) groups like water, proteins, etc., [27]. This is due to the high-energy vibrational frequency of -OH which increases the non-radiative relaxation of the excited states of the lanthanides. Bogdan et al. [28] reported the quenching of Er^3+^ ions with -OH groups due to multiphonon relaxation of the ^4^I_11/2_→^4^I_13/2_ and ^2^H_11/2_/^4^S_3/2_→^4^F_9/2_ transitions. They also mentioned that the relaxation pathways favored the formation of ^4^F_9/2_ states with red emission that occurred at the expense of the green luminescence. The requirements of high laser power and heating effects also limit the use of UCNPs to a greater extent. While polymeric nanofibers can be used to harbor the UCNPs to monitor (bio)analytical processes, on the other hand, it remains a challenge to integrate functionalized nanofibers within a microfluidic platform. The main advantage of incorporating the nanofibers is to create more surface area and porosity for immobilizing the recognition moieties for the detection of analyte. The integration of nanofibers into microfluidics has an edge over macro-electrode biomedical devices as the small geometry of a microfluidics chip provides better mass transport and diffusion, a low detection limit, and high signal-to-noise ratio [29]. The detection method based on emission luminescence of the UCNPs can provide a meaningful readout of the desired analysis. The effective synthesis of beta-phase NaYF_4_:20% Yb^3+^, 2% Er^3+^ nanocrystals with controllable dimensions ranging from 4.5 to 15 nm was demonstrated by Cohen and colleagues. They optimized the temperature, ratio of Y^3+^ to F^−^ ions, concentration of basic surfactants, and other test variables to modify the dimensions of the UCNPs [30]. Lanthanide-doped NaYF_4_ nanoparticles with governed crystal phase and peak performance of upconversion luminosity with thermal breakdown were demonstrated by Milliron and colleagues using an automated platform [31]. Hence, we could say that by adjusting and monitoring the test variables of the UCNPs and polymeric nanofiber, one can synthesize UCNP@nanofibers of variable sizes and shapes, such as cylinder, beads-on-string, and ribbon [11]. Large-scale synthesis of pure, super-long, single-crystalline YAG nanofibers using one-step calcination has also been reported wherein enhanced Photoluminescence (PL) from solitary YAG:Eu^3+^ electrospun nanofibers was influenced by Eu^3+^ ions; this polarized PL also depended on the dimension of the nanofibers [32].

### 1.2. Impact of Design of UCNP@nanofibers on Luminescence Efficiency

Pang and his colleagues [33] incorporated hydrophilic NaYF_4_:Yb^3+^,Er^3+^ Nanoparticles (UCNP-COOH) into photoluminescent nylon 6 (PA6)/PMMA nanofiber using co-electrospinning and spin-coating processes. The transparent UCNP-COOH/PA6/PMMA nanofiber mats exhibited strong green and red upconverted emission under 980 nm laser excitation, and the upconversion could be tuned by adjusting the weight fraction of the nanoparticles. Two green and one red emission band of Er^3+^ were observed at 521 nm (^2^H_11/2_→^4^I_15/2_), 539 nm (^4^S_3/2_→^4^I_15/2_), and 654 nm (^4^F_9/2_→^4^I_15/2_). However, the upconverted emission decreased slightly in the polymeric PMMA matrix due to the slow relaxation processes [34]. When NaYF_4_:Yb^3+^,Er^3+^ was electrospun in PVP nanotubes, significant upconversion luminescence was observed [35].

**Figure 3:**
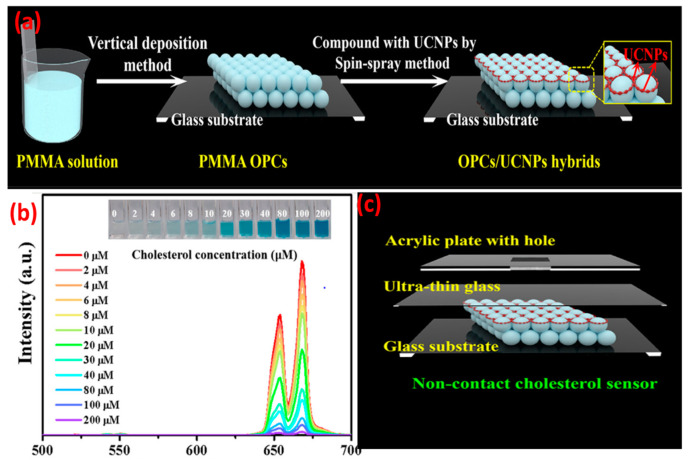
(**a**) Formation process for OPCs/UCNPs; (**b**) UC emission change of LiErF_4_:0.5%Tm^3+^@LiYF_4_ UCNPs versus concentration of cholesterol (2 to 200 μM) under 980 nm excitation. (**c**) Construction of a non-contact OPCs/UCNPs cholesterol sensor. The inset shows the corresponding photographs of the colored products. (Adapted from Ref. [40]).

When compared with NaYF_4_:Yb^3+^, Er^3+^ nanoparticles, the UCNPs@PVP nanofibers showed enhanced violet (381 nm), blue (409 nm), green (520 and 541 nm), and red (653 nm) emissions of Er^3+^. In the case of UCNPs, presence of non-radiative centers on the surface and high-phonon-energy groups would transfer the energy to non-radiative centers and enhance the non-radiation relaxation. Presence of PVP on the UCNPs could effectively eliminate such energy surface traps and suppress the quenching of non-radiative pathways. Concentration of activators in UCNPs has a significant effect on upconversion efficiency. Lahtinen and colleagues [36] studied the effects of varying doping percentages of Tm^3+^ and Er^3+^ on luminescence intensity. NaYF_4_:Yb^3+^ with Er^3+^ (3 and 20% doping) and NaYF_4_:Yb^3+^ with Tm^3+^ (0.5 and 8% doping) were selected for measuring the brightness and decay behavior of upconversion at high excitation intensity. NaYF_4_:Yb^3+^ with 8% Tm^3+^ favored ^1^D_2_→^3^F_4_ transition in the blue spectral region with an emission at 450 nm. Higher Tm doping also shortened the luminescence decay time to 31 μs. UCNPs with lightly (3%) and highly (20%) doped Er^3+^ activator ions exhibited a shorter decay time and emissions at 550 nm (^4^S_3/2_→^4^I_15/2_ transition). However, higher doping of Er^3+^ did not show significant enhancement in the emission intensity. There has been a growing interest in using UCNPs in (bio)analytical applications, for example, to monitor the redox state of an enzyme. Oakland et al. [37] monitored the presence of a flavoprotein, pentaerythritol tetranitrate reductase (PETNR), by detecting the variation in energy transfer from the UCNPs (Tm-based) to the flavin cofactor (flavin mononucleotide) of the redox state of PETNR. The emission at 475 nm (^1^G_4_→^3^H_6_) was quenched by the enzyme. By altering the rare-earth dopant, the emission profile of the UCNPs can be tuned to monitor biological molecules, e.g., glucose oxidase, vitamin B12, and heme cofactor of cytochrome c. However, it is also important to maximize the apparent energy transfer (AET) between the donor UCNPs and the acceptor biomolecules. This can be achieved by attaching the biomolecules to the surface of the UCNPs and placing the donor–acceptor moieties within the Förster radius. Rare-earth upconversion phosphors (UCPs) were also used to probe the redox chemistry of PETNR [38] wherein the changes in FRET between the emission band of the UCPs and the absorbance band of the enzyme was monitored. With a continuous wave excitation at 980 nm, the UCPs showed two emission bands in the blue (475 nm; ^1^G_4_→^3^H_6_) and near IR regions’ (800 nm; ^3^H_4_→^3^H_6_) transitions of Tm^3+^. The separation of the transition bands (~500 nm) allowed for a ratiometric analysis of the enzyme concentration by monitoring the variation in the ratio of the two emission bands. The spectral multiplexing capability of UCNP-QDs was used for the recognition of biotin-streptavidin in a competitive replacement assay. Due to the spectral overlap of QDs FRET acceptor with the UCNPs donor, the PL quantum yield was enhanced [39]. The FRET process was effective because both the donor and acceptor were fluorescent and the distance between them was <10 nm. The study opened the possibility of developing upconverted luminescent probes for the ratiometric detection of enzyme–substrate metabolism. Zhang et al. [40] modified the immobilization technique in which, firstly, the detection solution containing the enzyme (cholesterol oxidase) and different concentrations of cholesterol were mixed and incubated for 30 min at 37 °C. The cholesterol was catalyzed with the enzyme to produce H_2_O_2_ and choleste-4-en-3-one. The generated H_2_O_2_ oxidized 3,3′,5,5′-tetramethylbenzidine (TMB) in the presence of HRP to form the oxidation products (Ox-TMB) which showed intense absorption at ~652 nm that overlapped with the red upconversion of LiErF_4_:0.5%Tm^3+^@LiYF_4_ UCNPs. The detection mixture was dropped into an ultrathin glass sheet containing the UCNPs-and-PMMA-based opal photonic crystal. The monochromic red UC emission of the UCNPs were quenched with Ox-TMB, and the intensity of the quenching was proportional to the cholesterol concentration (Figure 3).

## 2. Trends in Embedding UCNP’s in Nanofiber

In addition to natural and synthetic polymers, nanofibers can also be synthesized using ceramic, carbon-based, and semiconducting nanomaterials. Because of their wide diameter range (10–1000 nm), controlled pore size, and potential for use in bio(analytical) devices, polymeric nanofibers have drawn the most attention among various types of nanofiber materials. Some standard spinning methods for synthesizing polymeric nanofibers include melt-blowing, centrifugal spinning, electrospinning, template synthesis, self-assembly, phase separation, and melt spinning. Innovative approaches have been focused on creating reliable and affordable spinning methods, and making them more scalable and easier to use. Considering this, further cutting-edge techniques for the creation of nanofibers have been presented, including solution-blown spinning, CO_2_ laser supersonic drawing, airflow bubble spinning, plasma-induced synthesis, and electro-blown spinning [41].

### 2.1. Recent Techniques Used for Embedding UCNPs into Nanofiber

We have explained a few techniques briefly in the following section.

Airflow bubble spinning: Liu and his colleagues fabricated highly oriented PLA nanofibers with nanoporous structures by processing variables of solution concentration and airflow temperature, and collecting distance and relative humidity. The innovative method effectively eliminates the possible safety risks brought about by unanticipated static electricity to manufacture highly orientated nanoporous fibers [42].

Plasma-induced synthesis: CuO and ZnO nanoparticles were formed by bombarding the electrodes’ surface rapidly with energetic radicals, subsequently followed by atom vapor diffusion, plasma development, solution medium water retention, in situ oxygen response, and additional growth. Hu et al. synthesized fiber-shaped cupric oxide (CuO) nanoparticles and flower-shaped ZnO nanoparticles using a plasma-induced technique directly from a copper and zinc electrode pair in water [43].

CO_2_ laser supersonic drawing: To create nylon 66 nanofibers, as-spun nylon 66 fibers have been drawn at supersonic speeds and then subjected to radiation from a carbon dioxide (CO_2_) laser. Using the fiber injector orifice, air was thrust into a vacuum chamber resulting in a supersonic jet. A nanofiber with an average diameter of 0.337 μm and a draw ratio of 291,664 was achieved at a laser power of 20 W and a chamber pressure of 20 kPa. Its drawing speed in the CO_2_ laser supersonic drawing was 486 ms^−1^. Two melting peaks were visible in the nanofibers at roughly 257 °C and 272 °C. The lower melting peak is observed at the same temperature as that of the as-spun fiber, whereas the higher melting peak is about 15 °C higher than the lower one [44].

The above techniques can be used for synthesizing UCNPs’ embedded nanofibers which can be used in (bio)analytical devices. As electrospinning is one of the most cost effective and flexible approaches, we are focusing this review specifically on the trends of UCNP@nanofibers synthesized using electrospinning techniques. These techniques have an assortment of perks, which involve the need for effortless, cost-effective furnishings, an outstanding rate of success, the capacity to track the NFs’ morphology, and the practical use of most polymers—even those with large molecular weights [45].

### 2.2. Advantages of Entrapment of Upconversion Nanoparticles inside Nanofibers

While on one hand, nanofibers provide an anchoring platform and stability to the nanoparticles, on the other hand, nanoparticles provide functionalities to the nanofibers. Therefore, the combination of both constituents gives rise to a newer material that has the advantages of both the components or the synergistic effect from both components. In the present scenario, the development of a single multifunctional platform is in demand in almost every field of application like catalysis, drug delivery, biosensors, bio-imaging, and energy devices. The incorporation of functional nanoparticles into electrospun nanofibers provides improved chemical, electrochemical, mechanical, and optical performances. However, tuning of the material’s structure and its function is crucial in developing an effective single multifunctional platform. The porosity of the nanofibers allows for embedment of a substantial amount of guest molecules on their large surface area and helps in their retention within the nanofibers [46,47]. The field of UCNPs research makes advances on various fronts including chemical and optical stability, and narrow-band-gap emission. Most recent research into the development of the UCNP@nanofiber composite material has demonstrated that nanofibers can provide solutions to the obstacles the mere nanoparticles themselves cannot overcome. This includes protection from the destructive influence of molecular oxygen [48], protection from water quenching exhibiting a 50-fold increase in luminescence intensity [49], and negation of any problem with colloidal stability as the UCNPs are distributed wherever nanofibers are placed. Yet, doping nanofibers with UCNPs changes the nanofiber fabrication conditions [50], which need to be thoroughly investigated and possible light scattering of the fibrous mats must be considered for bioimaging and quantitative analyses [51].

The following section focuses on the inherent advantages demonstrated for quantitative analyses through the integration of UCNPs into nanofibers. UCNPs have been successfully incorporated into electrospun nanofibers made up of polymers (e.g., PMMA, polystyrene, PVP, poly(e-caprolactone)) and demonstrated that unique properties of this composite material enable biomedical in vivo detection and (bio)analytical sensor applications [52]. Specifically, Baeumner and colleagues [49] reported that core–shell NaYF_4_(20%Yb, 2%Er, 10%Gd)@NaYF_4_ electrospun PVP nanofibers in a microfluidic channel could overcome limitations like the colloidal stability of the nanoparticles, photo-bleaching of the UCNPs, and high background signals. The porous nanofiber mat helped in retaining the luminescence intensity of UCNPs@nanofibers that was unaltered by changes in buffer or pH, and hence, the in situ generated light onside the platform could be used for sensing or as a light source for photoreactions. Similarly, Song et al. [53] reported the retention of photophysical properties of UNCPs inside a composite nanofiber material. The photoluminescent UCNPs@nanofibers is envisaged to provide insights into biological mechanisms and metabolic pathways as they can serve, unlike traditional imaging tools, as label-free sensitive tools for the detection of changes in metabolic activities in vivo, with high resolution and sensitivity [54]. The composite material can also be used as trigger for drug-release, or as activators in photo-dynamic therapy [55,56].

Zhang et al. [57] reported electrospinning of hydrophilic core–shell β-NaYF_4_:Yb,Tm@NaYF_4_ nanoparticles (containing 30% Yb^3+^ and 0.5% Tm^3+^) in CdS/PVP/TBT nanofiber mats. The average diameter of the nanofibers was 600 nm in which UCNPs and CdS were embedded. The UCNPs@nanofibers demonstrated good photocatalytic degradation of Rhodium B dyes and the authors envisaged the potential of the UCNPs@nanofibers for applications in solar cells, bio-imaging, and PDT. Along similar lines, Y_2_Ti_2_O_7_:Tm^3+^/Yb^3+^ nanoparticles were electrospun into nanofibers which, on irradiation with 980 nm laser, showed prominent emissions at 480, 656, 689, and 803 nm assigned to the Tm^3+^ transitions of ^1^G_4_→^3^H_6_, ^1^G_4_→^3^F_4_, ^2^F_2_,_3_→^3^H_6_, and ^3^H_4_→^3^H_6_. An increase in the emission intensity was observed with an increase in Yb^3+^ concentration; however, their shape and positions remained the same [58]. Maria et al. [59] reviewed the applications of lanthanide doped UCNPs for physical sensing by monitoring the change in the photoluminescence of the nanoparticles. Hirsch and colleagues [60] developed a self-assembled Tm^3+^-doped NaYF_4_ and gold nanotriangle for NIR to UV upconversion, and it was used for sensing Vitamin B12 in the serum. The plasmonic properties and geometry of the nanoparticles played an important role in emission enhancement by creating localized surface plasmons. Peng et al. [61] used homogenous core–shell NaYF_4_:Yb/Tm@NaYF_4_ upconversion nanocrystals as energy donors to a dye receptor for sensitive and rapid monitoring of Zn^2+^ ions. The sensing platform (PAA-UCNPs) showed blue emissions of Tm^3+^ at 475 nm under excitation at 980 nm in the absence of Zn^2+^. The upconversion luminescence was quenched in the presence of Zn^2+^ with a blue-shift of the band from 475 to 360 nm. Recently, Gu and Zhang [62] provided a comprehensive review on UCNP-based biodetections of inorganic ions, gas molecules, reactive oxygen species, thiols, and hydrogen sulfide. Generally, biological macromolecules like enzymes, proteins, DNA, aptamers, and yeast are immobilized onto nanofibers with physical (embedded or adsorbed) or chemical (cross-linked or covalently bonded) interactions [63] to prevent degradation and retain metabolic efficiency in biological activities [64]. An electrospun poly(methyl methacrylate)/polyaniline material nanofiber was used for the effective immobilization of laccase through both adsorption and covalent binding [65]. The relatively high enzyme immobilizations (110 mg/g and 185 mg/g of laccase attached using adsorption and covalent binding, respectively) were attributed to the fiber porosity and functional groups present on it. Both the systems showed retention of 80% of catalytic activity after one month and after 10 consecutive cycles that proved the stability of the enzymes within the nanofibers. Likewise, nanofibers of terpolymer poly (glycidyl methacrylate-co-methylacrylate)-g-polyethylene oxide containing reactive epoxy groups and a hydrophilic polyethylene oxide branch chain were used to covalently bind lipase for providing good thermal and organic solvent stability to the enzyme [66]. Nylon 6,6 nanofibers were used to embed multiwalled carbon nanotubes for developing an immobilization matrix for glucose. The presence of free aldehyde groups on the polymeric surface provided attachment sites for the enzyme using covalent bonds. The nanofibrous platform was made electroactive by coating it with a conducting polymer,(poly-4-(4,7-di(thiophen-2-yl)-1H-benzo[d]imidazol-2-yl)benzaldehyde) wherein immobilization was enhanced due to the large surface area of the nanofibers [67]. Thus, besides providing stability to biological macromolecules against thermal, solvent, proteolytic attack, and other denaturing agents, the nanofibers also provide better control over enzymatic reactions and a platform for embedding multiple enzymes or different admixture molecules [68,69]. Multiple emission bands of UCNPs can be used to design ratiometric enzymatic sensors in which one emission band can be used as a reference and the other can be indicative of the analyte variation [70]. There are two ways in which emission can be modulated: firstly, through the selection of specific biorecognition units (indicator) whose absorption band specifically overlaps the emission band of the UCNPs (not the reference band), and secondly, by monitoring the changes in the emission intensity of the UCNPs in the presence of analyte [71,72]. Ni et al. [73] developed an optical probe using β-NaYF_4_:Yb^3+^/Er^3+^ nanoparticles for sensing cysteine in an aqueous solution. The UCNPs underwent the FRET process, in which an energy transfer took place from the donor UCNPs to an acceptor dye molecule (Rhodamine). A variation in emission spectra of UCNPs is seen in which the green emission intensity at 521 nm and 540 nm decreased with the increasing concentration of cysteine. No change in the red emission at 651 nm was observed because it does not overlap with the absorption band of the dye and hence is used as an internal reference.

**Figure 4:**
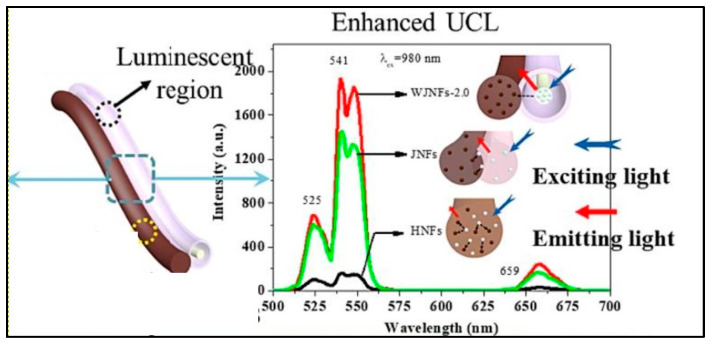
Enhanced PL (Phololuminescence) of UCNP@Nanofiber ([YF^3+^:Yb^3+^, Er^3+^@SiO_2_]//CoFe_2_O_4_ WJNFs) (Adapted from [74]).

In a work, Sheng et al. created a novel, unusual, 1D wire-in-tube nanofiber, called a Janus nanofiber (WJNF), wherein [YF^3+^:Yb^3+^, Er^3+^@SiO_2_]/CoFe_2_O_4_ was electrospun parallelly to the nanofiber. Simultaneous synthesis of SiO_2_, rare earth fluoride, wire-in-tube nanofibers, Janus nanofibers, and magnetic CoFe_2_O_4_ was achieved without the need for any extra protective gases. The in situ synthesis of unique Janus nanofibers via a one-pot mode offers the avoidance of post-processing procedures, and significantly improved the luminescence property (Figure 4) [74].

The trend to embed fluorescent or upconverted nanoparticles onto the enzyme immobilized nanofiber for the monitoring of enzymatic activities is on rise. A change in the luminescence emission or quenching of the fluorescent or upconverted nanoparticles on exposure to varying physical or chemical environments forms the basis of sensing or imaging. Thus, once the nanoparticles are embedded or attached to the enzyme mobilized nanofibers, they contribute to a highly sensitive and specific sensing platform due to the specificity of the analyte recognition unit. Functionalization of the nanofibers with recognition units can synergize the luminescence response and ratiometric sensing of analytes. However, assembling the UCNPs on the functionalized nanofibers (containing specific enzymes, proteins, antibodies, etc.) and optimization of the sensing layer are the challenges of an electrospun nanofiber. A major advantage of an electrospun-based sensing platform is that, on one hand, the nanofibers can be easily functionalized with bio-recognition units like enzyme, proteins, etc., using conventional coupling methods, while on the other hand, the nanoparticles can be directly incorporated into spinning solution. Therefore, the functionalization method becomes easy and post-processing steps can be avoided. Further, the bio-recognition molecules and nanoparticles could retain their functions and demonstrate better stability. Progress has been made in increasing the luminescence of the UCNPs embedded in the nanofibers.

### 2.3. UCNP@nanofibers as Sensing Platform for Bio(analytical) Assays

**Figure 5:**
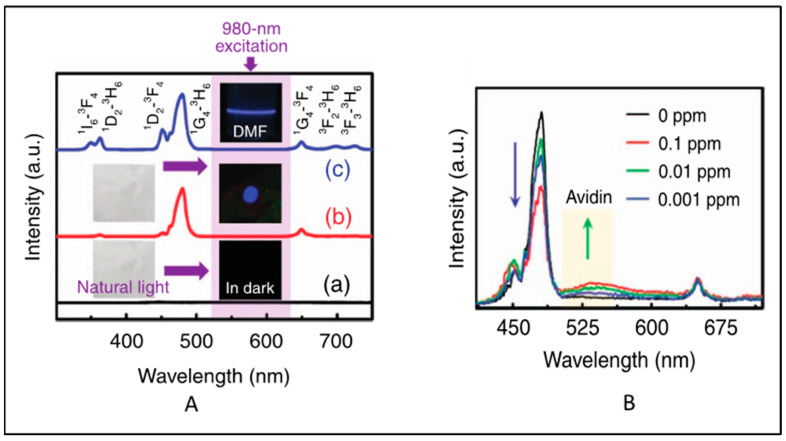
(**A**) Standardized UC emission spectra of (a) PS nanofibrous membrane (b) the NaYF_4_:Yb^3+^,Tm^3+^/PS HFM and (c) the NaYF_4_:Yb^3+^,Tm^3+^ NPs dispersed in DMF solution. The insets show the corresponding optical images obtained under natural-light or 980 nm LD irradiation. (**B**) Normalized UC emission spectra of the NaYF_4_:Yb^3+^,Tm^3+^/PS HFM loaded with a single water droplet containing different concentrations of avidin (Adapted from Refs. [78,79]).

This section focus on the latest research on functionalized electrospun nanofibers containing signal-generating luminescent moieties. UCNPs were used as an NIR-fluorescent-monitoring probe for non-invasive tracking of the degradation of hydrogel [75]. In this work, the polydopamine (PDA)-coated yolk−shell nanoparticles NaGdF_4_:Yb^3+^,Er^3+^@NaGdF_4_@PDA were cross-linked with carboxymethyl chitosan and sodium alginate hydrogel, and the enzymatic degradation of the hydrogel in lysozyme was monitored using the fluorescence intensity of the UCNPs. In 2018, Yao et al. [76] reported a color-tunable CdTe-QDs-based luminescent nanofibers for glucose sensing. Core–shell UCNPs (NaYF_4_:Yb^3+^/Er^3+^@NaYF_4_) in PMMA nanofibers exhibited luminescence at 540 (^4^S_3/2_→^4^I_15/2_) nm and 660 nm (^4^F_9/2_→^4^I_15/2_) upon pulsed near-infrared excitation [77]. Yb^3+^/Tm^3+^and Yb^3+^/Er^3+^ co-doped NaYF_4_ nanoparticle/polystyrene (PS) hybrid free-standing nanofiber was used as a fluorescence sensor for detecting avidin from a single water droplet [78]. No upconversion was observed in pure PS films. After incorporation of the UCNPs into the PS nanofibers, strong blue UCL (^1^G_4_→^3^H_6_ transition of Tm^3+^) was observed upon 980 nm excitation (Figure 5A). However, the intensity ratio of pure NaYF_4_:Yb^3+^, Tm^3+^ NPs decreased after embedding in the PS nanofibers. This may be due to the fact that some vibrational transitions of PS may have interacted with the excited levels of NaYF_4_:Yb^3+^, Tm^3+^ NPs and caused a nonradiative energy transfer process. However, the intensity of NaYF_4_:Yb^3+^, Er^3+^ NPs were significantly enhanced after embedding within PS. This may be due to the effective suppression of the thermal effect caused by laser excitation and the cross-relaxation phenomenon [79]. When avidin droplet-loaded NaYF_4_:Yb^3+^, Tm^3+^/PS HFM was excited with a 980 nm laser, an emission band at 530 nm emerged, which increased with the increase in avidin concentration. However, the blue Tm^3+^ band at 480 nm decreased with the increase in avidin concentration. This is attributed to the fluorescence group of avidin that adsorbed the blue photon and re-emitted a lower-energy green photon through a down-conversion luminescence process (Figure 5B).

By employing the electrospinning methodology, Ge et al. constructed pyrochlore phase Bi_2_Ti_2_O_7_ matrix nanofibers over an assortment of molar ratios (Tm^3+^/Yb^3+^ = 1:2–20) measuring at about tens of micrometres in their length and 200–300 nm in their diameter, producing PL emission bands in the range of 400–800 derived from the transitions of ^1^G_4_ → ^3^H_6_, ^1^G_4_ → ^3^F_4_, ^3^F_2,3_ → ^3^H_6,_ and ^3^H_4_ → ^3^H_6_, respectively. The particles had strong blue emission, and the temperature-sensing properties of Bi_2_Ti_2_O_7_:Tm^3+^/Yb^3+^ fibers (Tm^3+^/Yb^3+^ = 1:8 showed a relatively high sensitivity value of 0.024 K^−1^ at 300 K. The findings showed that for the purpose of detecting the surface temperature, the FIR approach had superior temperature-sensing capabilities over the thermal infrared imager. This finding will open up new avenues for the development of contactless FIR temperature-sensing applications of upconversion materials and spark a great deal of interest in upconversion studies in bismuth-based oxides [80].

**Figure 6:**
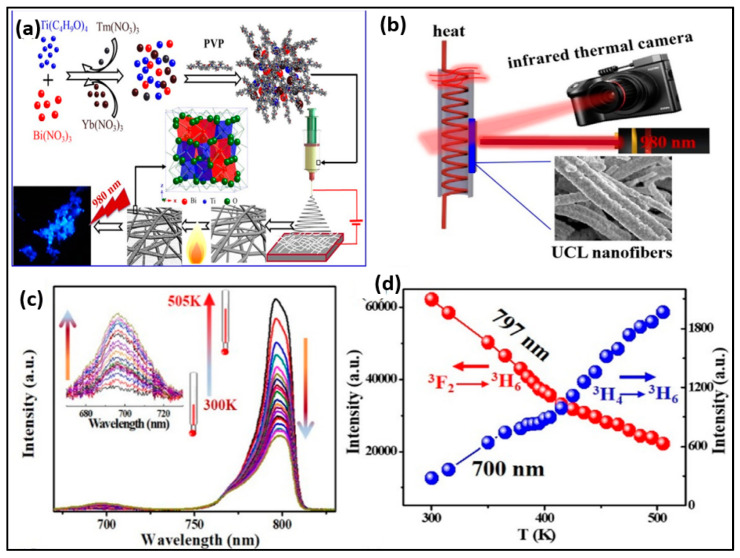
(**a**) Schematic showing mechanism of Bi_2_Ti_2_O_7_:Tm^3+^/Yb^3+^ upconversion nanofibers; (**b**) schematic diagram for the measurement process; (**c**) temperature-dependent spectra for Bi_2_Ti_2_O_7_:Tm^3+^/Yb^3+^(Tm^3+^/Yb^3+^ = 1:8); (**d**) luminescence intensity of the two thermal coupled levels (^3^H_4_, 797 nm; ^3^F_2,3_ 700 nm) at various temperature samples (Adapted from [80]).

Liu et al. synthesized lanthanide doped polyacrylonitrile (LOS-EY/PAN) nanofibers and proposed a dual temperature feedback fluorescence intensity ratio technique, which opened up the possibility of optical temperature sensing in micro-regions and created a ratiometric strategy for the excitation of Er^3+^ ions using a unique upconversion (UC) process. Cross-relaxation (CR) of Er^3+^ ion pairs have been defined as the odd three-photon illumination process implicated for both the red and green UC emissions of Er^3+^ single doping. Energy transfer from Yb^3+^ to Er^3+^ plays a dominant role, and the excitation procedure transforms into the traditional two-photon process. When Yb^3+^ comes into play, the CR process is compelled to pause, and energy is absorbed by Yb^3+^ as a result of its massive absorption cross-section contrasted to that of Er^3+^. With the reorganization of the excitation mechanism, the overall rise in Er^3+^ emission is followed by a boost in the aggregate quantum yield, which increases from 8.31 × 10^−5^ to 1.23 × 10^−3^. Relevant light stimulation enhances the proportion of signal to noise in usage and retains the degree of sensitivity at a high level of 1.29% K^−1^, proving the remarkable self-feedback nano-temperature-sensing function of LOS-EY/PAN fibers offer as an adaptive sensing component for fabrics and biomedical devices (Figure 6) [81]. Liu et al. prepared Gd_2_O_3_:Er^3+^@Gd_2_O_3_:Yb^3+^ core–shell nanofibers using electrospinning. Visible light upconversion photoluminescence reveals a prominent red emission band with distinct splitting peaks under 980 nm excitation, which is caused by a dramatic splitting of the energy level. The visible emissions exhibit temperature sensitivity within the 303–543 K range. The red emission demonstrates quenching with the rise in temperature. Thermal quenching had an activation energy of 0.1408 eV. The temperature-dependent multi-peaks of red emission were explored, and absolute sensitivity was obtained based on the valley and peak ratio of I680.31 nm/I683.03 nm in upconversion emission spectra. These findings imply that Gd_2_O_3_:Er^3+^@Gd_2_O_3_:Yb^3+^ nanofibers are good candidates for luminescence thermometry, which could lead to their use in both industry and scientific study [82]. Composite material nanofiber fabrication requires delicate circumstances for reaction; therefore, the outcome of the composite nanofiber’s dimensions can be difficult to control. Zhaoxue et al. synthesized the NaYF_4_ upconversion material, which is composed of Yb^3+^ as well as Er^3+^ rare earth ions. Subsequently, a single-stage electrospinning methodology has been employed for encasing the upconversion radiant NaYF_4_: Yb^3+^, Er^3+^ nanoparticles (NaYF_4_ NPs) beneath poly(lactide-co-glycolide)-gelatin (NaYF_4_-PLGA-gelatin). A detailed analysis was conducted on the impact of NaYF_4_ NPs on the mechanical properties, hydrophilicity, upconversion emission spectra, morphology, and degradation of the electrospun NaYF_4_-PLGA-gelatin nanofiber. When 5 mg/mL of NaYF_4_ NPs are encapsulated, the electrospun NaYF_4_-PLGA-gelatin nanofiber exhibited maximum luminous intensity. When compared to nanofibers with various concentrations, the mechanical properties of those with this encapsulated content are likewise often higher. Furthermore, a range of NaYF_4_ NP concentrations in the electrospun NaYF_4_-PLGA-gelatin nanofibers exhibit excellent hydrophilicity and degradation rates [83]. In a study, Ag@SiO_2_ and upconversion nanorods were electrospun into the nanofibers to create filamentous signaling sheets with superior upconversion luminescence, extremely high hydrophobic material, and adaptability. Riboflavin and pH were independently detected in human bloodstreams using the nanofibers. Using a single droplet, the fibrous signal material can identify levels as low as 0.01 ppm riboflavin and 0.1 pH. The droplet of material can be readily ejected after recognition, allowing for great reusability without impacting detection efficiency [84].

Table 1 presents an overview of various types of polymer that are used to embed UCNPs to form nanofibers.

## 3. Challenges in Embedding UCNP’s in Nanofiber

**Figure 7:**
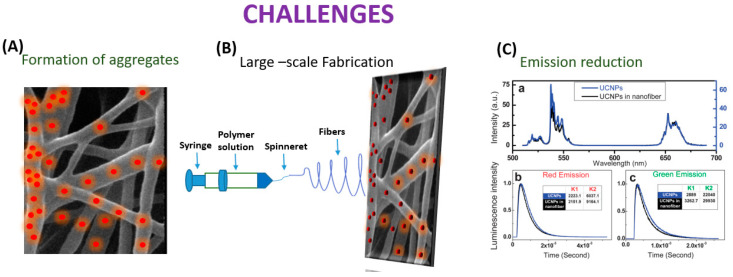
Schematic diagram depicting challenges in embedding UCNP’s in nanofiber.

Even though the process of electrospinning UCNPs into nanofibers has advanced significantly, there is still an assortment of areas that need to be improved (Figure 7).

In the electrospinning process, UCNPs have the potential to coalesce while UCNP@ nanofibers are being spun. According to Bao et al.’s research, the anisotropic propensity of the UCNPs to coalesce into particle chains near the hexagonal face was most likely caused by the non-polar hydrocarbon tails of the oleate capping ligands maximizing surface-area contact and van der Waals interaction. By using novel electrospinning techniques like coaxial, which provide more control over the orientation of the electrospun nanofibers, this constraint has been lessened. Additionally, Bao discussed the UCNP/PMMA nanofibrous films, and the UCNPs’ powder’s green emission to red emission luminescence intensity ratio (IG/R) in the spectra of the UCNP/PMMA nanofibrous films was lower than in the spectra from the powdered UCNPs. The process of creating sensors using electrospinning is fraught with difficulties. The primary goal is to create a homogenous, spinnable mixture. The consistency of the mixture and the proportions of the components are vital in this instance [85]. RE ions in matrix forms of polymers typically demonstrate relatively low emission efficiency, but effective fabrication of the crystal fiber via electrospinning is not doable, as demonstrated by Toncelli et al.’s review studies. On the other hand, when growing oxide crystal nanofibers, a mixture with the appropriate viscosity for the method can be acquired through the application of a polymeric precursor (which is typically PVP).

The material can then be eliminated through the ensuing calcination procedure, which can occur at conditions lower than those usually necessary for the stable state development of the crystal host. Following the calcination procedure, a further fluorination step is required for the development of fluoride crystal nanofibers. This makes the process more difficult because hazardous reagents are used in the fluorination process. Because of this, incorporating fluoride crystal nanoparticles into polymer fibers is another well-liked method. Fluoride materials are widely used as bulk crystal hosts and as nanoparticles. However, due to the relatively small number of published papers regarding oxide materials, the electrospinning growth of fluoride fibers is still in its early stages. When formed in bulk crystal form, fluoride materials need very high purity of the starting materials with careful management of the growing environment since even very low levels of contaminants greatly impair the emission efficiency of rare earth ions. This kind of material is typically generated using electrospinning and fluorinating oxide electrospun fibers. Bypassing the inherent challenges in the electrospinning growth of fluoride crystal matrixes, the bottom-up growth of nanoparticles has been optimized to produce high-quality monodisperse nanoparticles. Adding these high-quality nanoparticles to polymeric fibers is likely the simplest method to produce highly efficient upconverting nanofibers. To regulate the quality and shape of the materials developed, several other growth factors, such as the composition of the starting solution, the flow rate, the voltage, and the distance between the needle and collector, must be optimized. Moreover, the collector temperature and atmosphere humidity can have a large influence on the fiber quality. The electrospinning technique naturally leads to the growth of amorphous materials; therefore, a calcination step is always needed to obtain crystal nanofibers [86]. Among the obstacles and difficulties is the complexity of the fabrication process, the range of materials that can be spun, the homogeneity of the fibers, and reproducibility. Moreover, one of the present difficulties that researchers hope to solve in the future is the creation of portable sensors without the requirement for large apparatus. Additionally, it becomes difficult to quench and enhance the luminescence of UCNP@nanofiber. When UCNPs are embedded into nanofibers, aggregates may form, which could change the characteristics of both UCNPs and the nanofibers and limit their use in (bio)analytical devices as well as the needle–collector separation. Researchers should try to focus on the enhancement of the luminescence of the UCNP@nanofibers by changing the strategies of embedding lanthanide doped materials into the nanofibers. Enhancing UCNPs with multi-excitation wavelengths or improved UV emission is vital. Well-controlled nanostructures may also have greater potential to increase the energy transfer efficiency of UCNPs [87]. Despite the demands of industrialization, the large-scale production of UCNPs@nanofiber continues to be difficult. There are problems with jet interactions, needle clogging, and cleaning with multi-needle collaborative electrospinning. Initially, the needle-free electrospinning approach produces a lot of nanofibers, but their dispersion is incredibly uneven [88].

**Future perspectives:** Future research in replacing time-consuming analytical monitoring methods with a real-time monitoring system that is quick, sensitive, and efficient has been realized and found to be important in designing a (bio)analytical monitoring system. UCNPs have emerged in applications of biochemical sensors due to their unique and extraordinary optical and chemical features of having a high quantum yield, high resistance to photo-bleaching, low toxicity, long-term lifetime, narrow emission bandwidths, as well as very low optical background noise. Integration of UCNPs@nanofibers into microfluidic systems using bacterial cellulose nanopaper and chitin nanofibers papers [89] has opened an era of rapid and sensitive analyses of analytes using the luminescence of the nanoparticles. This new class of nanosensor can also be used to monitor dynamic processes at the molecular level upon interaction with NIR light, which is often inaccessible using other techniques. Analytical probes based on UCNPs can be used in both solution-phase and in heterogeneous assays and can, therefore, surpass conventional probes (like QDs, dyes, etc.). Heterogeneous assays based on UCNPs@nanofibers can be achieved using a microfluidic platform to fabricate point-of-care devices for on-site monitoring. Such a microfluidic platform can be devised if the UCNPs are of a smaller size, typically less than 10 nm, the synthesis of which is quite challenging. Further, absence of optical background interference in UCNPs, as opposed to other fluorescent molecules, can lead to the development of digital immunoassays where every UCNP can be counted as an optical probe. For that, it is important to synthesize the UCNPs of appropriate size, surface chemistry, and chemical entities (definite concentration/types of sensitizer and activators) and incorporate it in a suitable nanofiber matrix and ensure that they are bright enough to be detected at the single-nanoparticle level. Also, it is important to improve the UC emission efficiency and retain the same levels once it is inside the nanofibers. The upconversion emissions are adversely affected by the presence of surrounding molecules and, therefore, significant research is directed towards improving it, and the upconversion intensity and signal contrast [90]. UCNPs also provide a vehicle in sensing applications by emitting UV-Visible light and providing relevant signals for the measurements. Tsai and colleagues [91] used the green emission band of the UCNPs which was quenched with a pH-dependent inner filter effect (IFE), while the red emission band remained unchanged and acted as the reference signal for ratiometric pH measurements. Shen and colleagues [92] used biocompatible CaF_2_ to form an epitaxial shell on the UCNPs that showed high optical transparency and was effective in preserving the emission quenching in aqueous medium. The similarity in the lattice of CaF_2_ and NaYF_4_, a = 5.448 Å (CaF_2_, a = 5.451 Å) has paved the way in forming heterogeneous core–shell UCNPs that could provide resistance to aqueous quenchers and further research in this direction would lead to the use of UCNPs in photonics and biophotonics.

In this trend article, we have discussed UCNP-based nanofibers for the detection of analytes and their road to commercialization. A significant increase in sensitivity, ease of use, and easy fabrication techniques has added value to the miniaturized UCNP@nanofibers systems. The possibilities of integrating sensing transducers in microfluidic platforms and their smaller footprints can serve as important tool for evaluating bio(analytical) processes at the point of care. A microfluidic platform with a nanoengineered interface can serve as a sensor for several disease biomarkers. Though there are several benefits of an integrated microfluidics platform, commercialization of such systems may take a slightly longer time. One of the reasons may be since commercialization not only depends on innovations, but also on the feasibility of using the platform on a larger scale.

In our perspective, such systems could be the future of smart sensing technology; particularly when conjugated with a recognition moiety, for example an enzyme, sensors for specific analytes with low detection limit and specificity can be fabricated. However, such a device must be extremely robust and user-friendly. To be used for analytical applications, it is also important to investigate the sensitivity and multiplexing abilities of the UCNPs@nanofibers. For significant optimization, simple and economically feasible set ups are required for commercialization. For optimization, with respect to the activity and accessibility of the bioreceptor, binding events and signal transductions are required for accuracy, sensitivity, and selectivity of a bio(analytical) assay. The future perspectives of the development of a multiplexed microfluidic analytical system and the generation of organ-on-a-chip platforms are envisaged. An integration of bio-imaging and tissue engineering on a same platform may find applications in monitoring different organs and their responses to cellular parameters in situ, paving the way to advancing the field.

## Figures and Tables

**Table 1:** Various polymers used to embed UCNPs to form nanofibers and their applications.

Sr. No	Polymer	UCNPs	Applications	References
1	Polysulfone	LiYF_4_:Yb,Tm	Oxygen sensor	[20]
2	Biomolecules	Phosphor doped with Yb and Tm	Detection of presence of flavoprotein	[37]
3	PMMA	LiErF_4_:0.5%Tm@LiYF_4_	Detection of oxidase and cholestrol	[40]
4	PVP	NaYF_4_:Yb/Er and 10%Gd@NaYF_4_	Sensing the photoreaction	[49]
5	CdS/PVP/TBT	Β NaYF_4_:Yb/Tm@NaYF_4_	Degradation of rhodium B dyes	[57]
6	PAA	NaYF_4_:Yb/Tm@NaYF_4_	Monitoring of Zn	[61]
7	PDA	NaGdF_4_:Yb/Er@NaGdF_4_@PDA	Hydrogel degradation	[75]
8	PS	NaYF_4_:Yb/Tm and NaYF_4_:Yb/Er	Fluorescence sensor for detecting avidin	[78]
9	PAN	Gd_2_O_3_:Er^3+^@Gd_2_O_3_:Yb^3+^	Temperature sensing	[81]
10	PLGA	NaYF_4_:Yb^3+^, Er^3^	In vivo bioimaging	[83]
